# Regulation of the Wnt/β-Catenin Signaling Pathway by Human Papillomavirus E6 and E7 Oncoproteins

**DOI:** 10.3390/v7082842

**Published:** 2015-08-19

**Authors:** Jesus Omar Muñoz Bello, Leslie Olmedo Nieva, Adriana Contreras Paredes, Alma Mariana Fuentes Gonzalez, Leticia Rocha Zavaleta, Marcela Lizano

**Affiliations:** 1 Instituto de Investigaciones Biomédicas, Universidad Nacional Autónoma de México, Av. Universidad 3000, Col. Ciudad Universitaria, Del. Coyoacán, México DF CP. 04510, Mexico; E-Mails: omarmube@gmail.com (J.O.M.B.); alma_mariana2000@yahoo.com.mx (A.M.F.G.); lrochaz@biomedicas.unam.mx (L.R.Z.); 2 Facultad de Química, Universidad Nacional Autónoma de México, Av. Universidad 3000, Col. Ciudad Universitaria, Del. Coyoacán, México DF CP. 04510, Mexico; E-Mail: leslie_azul25@hotmail.com; 3 Unidad de Investigación Biomédica en Cáncer/Instituto Nacional de Cancerología-Instituto de Investigaciones Biomédicas, Universidad Nacional Autónoma de México, Av. San Fernando 22, Col. Sección XVI, Del. Tlalpan, México DF CP. 14080, Mexico; E-Mail: adrycont@yahoo.com.mx

**Keywords:** Wnt/β-catenin, HPV E6 and E7 oncoproteins, HPV-related cancers

## Abstract

Cell signaling pathways are the mechanisms by which cells transduce external stimuli, which control the transcription of genes, to regulate diverse biological effects. In cancer, distinct signaling pathways, such as the Wnt/β-catenin pathway, have been implicated in the deregulation of critical molecular processes that affect cell proliferation and differentiation. For example, changes in β-catenin localization have been identified in Human Papillomavirus (HPV)-related cancers as the lesion progresses. Specifically, β-catenin relocates from the membrane/cytoplasm to the nucleus, suggesting that this transcription regulator participates in cervical carcinogenesis. The E6 and E7 oncoproteins are responsible for the transforming activity of HPV, and some studies have implicated these viral oncoproteins in the regulation of the Wnt/β-catenin pathway. Nevertheless, new interactions of HPV oncoproteins with cellular proteins are emerging, and the study of the biological effects of such interactions will help to understand HPV-related carcinogenesis. This review addresses the accumulated evidence of the involvement of the HPV E6 and E7 oncoproteins in the activation of the Wnt/β-catenin pathway.

## 1. Introduction

Signaling pathways are the mechanisms by which cells decide their fate and communicate with other cells and their environment. The binding of ligands to cell receptors can activate protein cascades and consequently affect gene transcription levels. Via these complex processes, cells transform external stimuli into biochemical signals that control biological effects, such as proliferation, differentiation, and death.

Many signaling pathways have been identified as being deregulated in cancer. Consequently, numerous elements targeting these pathways have been proposed as therapeutic targets. Consistent alterations of some important pathways controlling cell proliferation and apoptosis, such as PI3K/Akt, ERK/MAPK, Notch, and Wnt/β-catenin, have been identified in different types of cancer. In particular, the activation of the Wnt signaling pathway has been implicated in osteosarcoma [1], hepatocellular carcinoma [2], colorectal cancer [3], and breast cancer [4]. More recently, this signaling pathway was also implicated in oral cavity, oropharyngeal [5], and cervical cancers [6,7].

Cervical cancer is the fourth most common cancer in women worldwide and is one of the leading causes of cancer death in women in developing countries [8]. Persistent infection with Human Papillomavirus (HPV) is a necessary factor for cervical cancer development [9]. HPV is also associated with other pathologies, such as head and neck [10] and anal cancers [11]. HPV types linked to cancer are those classified as high-risk HPV (HR-HPV), whose viral oncogenes interact and regulate the function of several cellular proteins.

This review addresses the participation of the Wnt/β-catenin signaling pathway in HPV-related cancers and the possible mechanisms by which HPV E6 and E7 oncoproteins induce the activation of this pathway.

## 2. Wnt/β-Catenin Cell Signaling Pathway

The Wnt signaling pathway is involved in development, proliferation [12], differentiation [13], adhesion [14], and cellular polarity [15]. The term Wnt, which was adopted in 1991, includes a family of genes that encode secretory glycoproteins. Wnt is an acronym of homologous wingless (wg) and Int-1, which had been described in the fly and mouse, respectively [16]. In 1982, Nusse and Varmus found that the mouse mammary tumor virus (MMTV) promotes mammary carcinogenesis in mice by inserting itself in a specific gene of the host genome [17]. They called this gene Int-1, and its nucleotide and amino acid sequences were obtained in 1984 [18]. Later, in 1987, the wingless gene in *Drosophila melanogaster* proved to be a homologue of Int-1 [19].

Currently, 11 receptors that are members of the Frizzled (Fz) family have been identified in humans. These receptors include Fz1 to Fz10 and Smo, as well as the two co-receptors LRP 5 and 6, and all of these receptors are responsible for Wnt signaling activation. Moreover, 19 Wnt ligands have been described for these receptors: Wnt1, 2, 2b, 3, 3a, 4, 5a, 5b, 6, 7a, 7b, 8a, 8b, 9a, 9b, 10a, 10b, 11, and 16 [20].

At least three signal transduction pathways activated by Wnt ligands are known, namely the canonical Wnt/β-catenin pathway and two non-canonical pathways: the planar cell polarity pathway (Wnt/PCP) and the Wnt/Ca^2+^ pathway. Moreover, the activation of the different pathways is ligand-specific, and the primary ligands that activate the canonical pathway are Wnt1, 2 [21], 3, 3a [22], 7a [23], 8 [24], and 10b [25,26]. The activation of the non-canonical pathways is mediated by Wnt4 [27], 5a [28,29], and 11 [30] ligands. However, diverse Wnt ligands have been shown to elicit various effects when binding to the same Fz receptor [31].

The non-canonical Wnt/PCP, also known as the Wnt/JNK pathway, is important in various processes including wound healing [32], the correct development of the neural tube [33], motility, and the modulation of cellular morphology [34]. These events are all generated by the reorganization of the actin cytoskeleton. Some of the main proteins involved in the transduction of the extracellular signal generated by Wnt/PCP are vangl2, celsr1-3 [35], Dvl, JNK, PKC [36], Rac, and RhoA [37].

In the Wnt/Ca^2+^ pathway, secondary messengers, such as IP3 and DAG, liberate calcium ions from the endoplasmic reticulum [29] and subsequently activate CaMKII [38] and PKC [39].

The processes that are triggered by the activation of this non-canonical pathway include the following: the regulation of convergent extension movements [40], the reorganization of the actin cytoskeleton [41], the modulation of cell motility [42], and the contribution to the inflammatory response [43].

**Figure 1:**
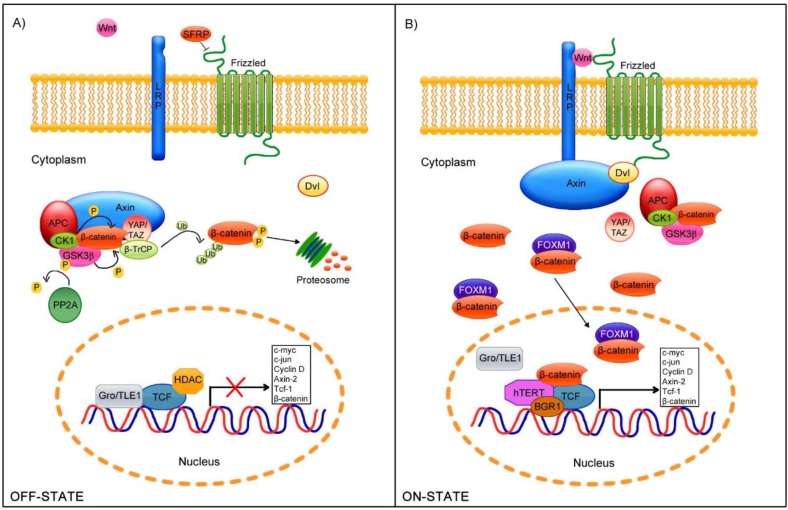
Wnt/β-catenin cell signaling pathway. (**A**) In the absence of stimuli (OFF-STATE), the Fz receptors are regulated by a group of antagonist proteins, such as SFRP, which prevent further receptor-ligand interaction. In the cytoplasm, a degradation complex is formed, to which β-catenin is recruited and phosphorylated at specific residues by the GSK3β and CK1 kinases. These phosphorylated sites are recognized by βTrCP ubiquitin ligase, which mediates β-catenin proteosomal degradation. In the nucleus, the Groucho/TLE repressor binds to TCF/LEF, avoiding its transcriptional activation; (**B**) In the presence of Wnt ligands (ON-STATE), LRP5/6 and Fz dimerize; subsequently, Axin binds to LRP5/6, whereas Disheveled (Dvl) interacts with Fz, allowing Axin-Dvl binding and the disassembly of the β-catenin degradation complex. Finally, β-catenin is released in the cytoplasm and translocated to the nucleus, aided by its binding partner FOXM1, where it binds to TCF/LEF and detaches the Groucho/TLE repressor.

The Wnt canonical signaling pathway is the best understood Wnt signaling cascade. In the absence of Wnt ligands (OFF-STATE), β-catenin is mainly located at cellular junctions. Nevertheless, a small amount remains in the cytoplasm and binds to a complex responsible for the degradation of β-catenin via the proteasome. This degradation complex consists of the scaffold protein Axin which recruits essential elements during this process such as GSK3β [44], CK1 [45], APC [46], YAP/TAZ, and β-TrCP [47]. CK1 phosphorylates β-catenin at the Ser45 residue, whereas GSK3β phosphorylates this protein at the Ser33, Ser37, and Thr41 residues [48,49]. Moreover, APC impedes the β-catenin dephosphorylation mediated by PP2A phosphatase [50]. Subsequently, the YAP/TAZ complex recruits the E3 ubiquitin ligase β-TrCP, which recognizes Ser/Thr phosphorylation, to promote β-catenin ubiquitination and its subsequent proteosomal degradation [47,51] (Figure 1A).

As a consequence of the Wnt ligand binding to the Fz receptor and LPR5/6 co-receptor [52] (ON-STATE), β-catenin delocalizes, accumulating in the cytoplasm [22] and nucleus [53,54]. When the Fz receptor dimerizes with the LRP5/6 co-receptor, the intracellular motifs of the Fz receptor recruits Disheveled (Dvl) protein [55], whereas CK1 phosphorylates LPR5/6 to allow Axin binding [56,57], which results in the disassembly of the β-catenin destruction complex. This process permits the accumulation and translocation of β-catenin to the nucleus. Moreover, the binding of FOXM1, a member of the Forkhead box (Fox) transcription factor family, to β-catenin promotes its nuclear translocation [58]. In the nucleus, β-catenin binds to transcriptional factors members of the TCF/LEF family [53,59], inducing the dissociation of co-repressors, such as Groucho/TLE [60], which allows the interaction with co-activators including CREPT [61], FHL2, and CBP/p300 [62,63] and remodelers of chromatin such as Brg-1 [64] (Figure 1B). These interactions with β-catenin promote the expression of diverse genes that regulate cellular polarity, proliferation, and differentiation, such as c-jun, c-myc, Cyclin D1, Axin-2, Tcf-1 [20], and β-catenin itself [65].

## 3. Human Papillomavirus

Persistent infection with Human Papillomaviruses (HPVs) has been implicated in the carcinogenesis of the uterine cervix [9]. Oropharyngeal [10] and anal cancers have also been related to HPV [11]. In fact, almost 70% of cervical cancer cases are associated with HPV16 and HPV18 [66].

The carcinogenic potential of HPV is mainly due to the expression of E6 and E7 viral proteins, which are directly involved in cellular transformation [67,68]. The E6 and E7 oncoproteins interfere with cell cycle regulators and induce genomic instability, which results in a malignant phenotype.

More than 170 HPV types have been identified [69]. HPVs can infect the differentiating squamous epithelium and are classified in two main groups: cutaneotropic and mucosotropic. The majority of cutaneous HPVs belong to the beta and gamma genus, whereas the alpha genus contains all known mucosal HPV types, and at least 40 members of this genus infect the anogenital region [70]. The mucosal HPVs are further divided according to the outcome of infection into low-risk HPVs (LR-HPVs), which are associated with benign and self-limiting benign warts, and high-risk HPVs (HR-HPVs), which are linked to pre-malignant lesions (low- and high-grade cervical intraepithelial neoplasia) and cancer. The most frequent HR-HPV types are: 16, 18, 58, 33, 45, 31, 52, 35, 59, 39, 51, and 56 [71].

Persistent HR-HPV infection is a crucial event in cellular transformation, but additional events are required to complete the malignant phenotype. Other mechanisms implicated in HPV-related cancers include the activation of multiple cellular pathways such as the Hedgehog [72], Erk/MAPK [73], Notch [74], and Wnt signaling pathways [75], which are involved in embryonic processes, differentiation, survival, proliferation, cell cycle progression, and self-renewal in stem cells.

### HPV Genome

The HPV genome consists of a double-stranded circular DNA of approximately 8000 bp that contains genes that are expressed early (E) or late (L) during the viral life cycle and whose transcription and replication are mediated by the long control region (LCR) [76].

L1 and L2 are the HPV structural proteins [77,78]. Specifically, L1 is the major capsid protein and constitutes approximately 80% of the viral capsid [77].

E1 is the viral DNA helicase, and E2 a transcriptional activator and repressor that also complexes with E1 as a critical component of the HPV replisome [79]. E2 protein plays a crucial role in the HPV life cycle due to its ability to regulate viral DNA replication and the transcription of E6 and E7 oncogenes [80].

The E4 coding sequence is contained within the E2 open reading frame (ORF). Although E4 is located in the early region, it is expressed as a late gene and is regulated by a promoter that is responsive to differentiation transcription factors. Moreover, the properties of E4 have not been fully characterized, but several studies implicate E4 in virion release via its association with keratin filaments [81].

E6 and E7 are considered the most important viral oncoproteins: they play a clear role in cellular transformation [82]. Among several cellular interactions, E6 oncoprotein binds to the tumor suppressor protein p53 and to the E3-ubiquitin ligase E6AP, promoting p53 degradation via the proteasome and facilitating DNA damage and mutation [83]. Furthermore, E7 oncoprotein associates with a complex that contains Cullin 2, an E2 ubiquitin ligase, leading to the degradation of the tumor suppressor pRB, to promote cell cycle progression [84].

During the normal viral life cycle, the HPV genome exists in host cells as an episome. However, the viral genome may be incorporated into the host genome in rare cases. Viral genome integration is closely tied to the development of cancer because most HPV-induced cervical cancer cases contain an integrated form of the HPV genome. Viral episome rupture during integration frequently occurs in a zone that includes E1 and E2. The consequent loss of E2 causes the uncontrolled expression of the E6 and E7 oncoproteins, which increases the likelihood of HPV-induced carcinogenesis [85,86].

## 4. E6 and E7 in Cellular Transformation

E6 and E7 are small proteins that localize to the nucleus and cytoplasm and the interaction of both E6 and E7 immortalizes primary cells in a highly efficient manner [87].

Moreover, the expression of E6 and E7 in organotypic raft cultures results in cellular changes that are similar to those observed in high-grade squamous intraepithelial lesions [88]. Accordingly, transgenic mice expressing HR-HPV E6 and E7 developed basal epithelial squamous carcinomas upon low-dose estrogen treatment [89]. In this model, E7 alone is sufficient to induce high-grade cervical lesions and invasive cervical neoplasia; nevertheless, the inclusion of E6 resulted in larger and more extended tumors. These data demonstrate the cooperative effect of E6 and E7 in promoting the development of cancer [67]. Even when E6 and E7 can immortalize cells in culture, these cells do not form tumors in nude mice models in which the co-expression of supplementary oncogenes, such as v-ras [90] or v-fos [91], is required for tumorigenesis [92].

During HPV infection, E6 and E7 induce the proliferation of undifferentiated and differentiated suprabasal cells and also inhibit apoptosis. These actions promote the accumulation of DNA damage and mutations that can result in cell transformation and the development of cancer [92]. Table 1 summarizes the known E6 and E7 cellular targets and their biological consequences.

**Table 1:** Cell biological effects induced by HPV E6 and E7 oncoproteins via interactions with cellular elements.

E6-Interactions	Biological Effects
Protein PDZ-domain	Degradation of proteins harboring PDZ domains, with a loss of cell architecture and polarity [93].
E6AP	Degradation of targets such as p53 [83]. Activation of hTERT transcription, inducing immortalization [94].
Bak, FADD Procaspase 8	Induction of respective protein degradation, suppressing apoptosis [95,96].
BRCA1	Activation of estrogen receptor ER signaling pathway [97].
Tyk2	Impairment of Tyk2 activation thereby inhibiting IFN-induced signaling [98].
CBP/p300	Down-regulation of p53 activity by targeting the transcriptional coactivator CBP [99].
NFX1-91	Degradation of NFX1-91 and activation of hTERT [100].
c-Myc	Increased hTERT gene expression [101].
Dvl2	β-catenin stabilization and Wnt signaling activation [102].
**E7-Interactions**	**Biological effects**
pRb family proteins	Disruption of pRb-E2F complexes thereby initiating the E2F-mediated transcription [103].
AP1	Transactivation of members of AP1 family [104].
Cyclin A/CDK2	Regulation of cell cycle [105].
Cyclin E/CDK2	Regulation of cell cycle (binding through p107) [106].
p21	Inactivation of p21, modulating CDK and PCNA inhibitory functions [107].
MPP2	Enhancement of MPP2-specific transcriptional activity [108].
p600	Contribution to anchorage-independent growth and transformation [109].
Mi2	Complexes with HDAC to promote the E2F2-mediated transcription [110].
IRF1	Abrogation of transactivation function of IRF1 [111].
p48	Down-regulation of IFN α-mediated signal transduction [112].
p27	Abolishment of p27’s cell cycle inhibitory function, which endows the cell with invasive properties [113].
PP2A	Inhibition of PP2A catalytic activity [114].

The E6 protein consists of approximately 150 amino acids and contains an LXLL motif in the amino terminal region that is required to interact with the ubiquitin ligase E6AP. Moreover, several proteins also bind to E6 via its LXLL motif, such as E6BP, IRF3, Tuberin, and Paxillin. Another critical E6 motif found in the carboxyl terminus is the S/TXV PBM (PDZ-binding motif), which mediates the interaction with specific domains on cellular proteins known as PDZ domains, specialized in protein-protein interactions [115]. E6 interactions with PDZ-containing proteins commonly induce their proteasome-mediated degradation [116]. PBM is present only in E6 of HR-HPV, suggesting a possible role for this motif in HPV-induced oncogenesis [117] (See Table 1).

The E7 protein consists of 98 amino acids separated in three conserved regions: CR1, CR2, and CR3. CR2 includes a conserved LXCXE motif that mediates high-affinity binding to pRB [118]. The CR3 region contains two CXXC motifs separated by 29 or 30 residues, forming a zinc-binding domain. This region is critical for interaction with cellular proteins, including pRB [119], p21 [107], p27 [120], TBP [121], and E2F [122] (See Table 1).

Growing evidence suggests that HPV oncoproteins can modulate cell signaling pathways to contribute to the carcinogenesis. Upon initial infection, this modulation may be necessary to complete the viral cell cycle and form infective viral particles. Nevertheless, the consistent high-level expression of viral oncoproteins may eventually alter the normal functions of the cell, triggering an uncontrolled transformation process. Via their different cellular interactions, E6 and E7 may be deregulating the different cell signaling pathways implicated in HPV-related cancers. Some of these pathways are involved in cell proliferation and apoptosis, such as PI3K/Akt, Ras/Raf, Notch, and Wnt/β-catenin [123].

## 5. Wnt/β-Catenin Signaling in HPV-Related Cancers

Several mutations in different components of the Wnt/β-catenin pathway have been described in various types of cancer [124]. In contrast, in HPV-related neoplasias, mutations in Wnt pathway members such as the CTNNB1 and AXIN1 genes are uncommon [125]. However, in cervical cancer biopsies and oropharyngeal squamous carcinoma cells, membrane β-catenin is lost, whereas cytoplasmic and nuclear β-catenin accumulation is observed during cancer progression [6,75]. Some studies have shown that LGR5, a member of the G protein-coupled receptor family, is progressively expressed in cervical neoplasia, promoting the proliferation and tumorigenesis of cervical cancer cells via the activation of the Wnt/β-catenin pathway [126]. Furthermore, post-transcriptional modifications have been identified in components that negatively regulate the pathway; for instance, in cervical cancer samples, GSK3β is inactivated by the phosphorylation of its Ser9 residue, inducing the over-activation of the Wnt signaling pathway [127].

Furthermore, patients with oral and lung cancers that express high levels of the β-catenin-binding partner FOXM1 exhibit worse overall and relapse-free survival than patients with tumors that express low levels of FOXM1; interestingly, this effect is significantly enhanced by the presence of HPV DNA sequences [128]. Therefore, alterations in Wnt cell signaling pathway regulatory elements are associated with cancer progression and poor prognosis in HPV-related cancers.

Epigenetic changes that suppress the activity of the negative regulator of the Wnt pathway have also been identified. Specifically, methylation markers in the APC and SFRP3 promoters have been found in ovarian cancer samples, but only in cases in which HR-HPV genomic sequences were detected [129]. Moreover, in cervical cancer samples, methylation markers have been found in the SRFP2 and DKK3 promoters [130].

Microarray expression studies of cervical cancer-derived tumors and cell lines have identified the over-expression of genes involved in Wnt pathway maintenance and regulation, such as JUN, MYC, FZD2, RAC1, GSK3β, Dvl-1, and CTNNB1 [131,132]. Specifically, Wnt/β-catenin elements are differentially expressed in HPV-positive cervical cancer cell lines (HeLa and SiHa) compared with a non-tumorigenic immortalized cell line (HaCaT) [133]. In this study, 38 genes were identified to be deregulated. Specifically, 15 genes were up-regulated (including CCND3, LRP5, TCF7, and FDZ9), and 23 gene were down-regulated (including CCND2, WNT10A, WNT7A, TCF3, WNT1, FZD4, and BTRC). Because these authors found that WNT7A expression was also significantly reduced in cervical cancer samples, they restored WNT7A expression in HeLa cells, which resulted in a strong decrease in cell viability, proliferation, and migration. In addition, aberrant hypermethylation in the CpG islands within the WNT7A promoter was found in HeLa and SiHa cells but not in HaCaT cells; this event suggests as a possible mechanism by which WNT7A is repressed.

Additionally, a systematic study in cervical cancer samples showed an alteration in the expression of miRNAs involved in Wnt/β-catenin pathway modulation [134]. In this study, miR-21-5p, miR-34c, and miR-96a were up-regulated, whereas miR-99b, miR-497-5p, and miR-617 were down-regulated. Although functional analysis was not performed, these expression patterns were hypothesized to modify the levels of their targets, *i.e.*, WNT5A, FZD1, FAS, MYC, FZD6, CCND1, and PDGFRA, to facilitate cell proliferation and invasion.

Clear evidence indicates that the Wnt pathway is hyperactivated in HPV-related cancers. Currently, HPV oncoproteins are known to bind and alter the function of several cellular targets associated with Wnt pathway regulation, including hTERT, p53, p300/CBP, Dvl, and PP2A (see Table 1), and information about the possible viral regulatory mechanisms in this pathway is emerging.

## 6. Wnt/β-Catenin Cell Signaling Regulation by E6 and E7 Oncoproteins

The activation of the canonical Wnt pathway represents a second requirement for the malignant transformation of the HPV-infected epithelium [75,135]. Specifically, several findings support the direct or indirect participation of HPV oncoproteins in this pathway.

In HPV-positive oropharyngeal cells, β-catenin expression is strongly localized in the cytoplasm and nucleus, whereas β-catenin is mainly detected in the membranes of HPV-negative cells [5].

In these HPV16-positive oropharyngeal cancer cell lines, E6 and E7 repression was shown to significantly decrease the β-catenin cytoplasmic and nuclear protein levels as well as the β-catenin mRNA levels. Moreover, both E6 and E7 expression were confirmed to up-regulate β-catenin expression and to enhance TCF-mediated transcription. This effect was attributed to a decrease in the ubiquitin ligase type 3 Siah-1 protein (seven in absentia homologue-1 protein), which acts as β-TrCP to induce β-catenin degradation. Because p53 mediates Siah-1 transcriptional activation [136], the down-regulation of p53 induces a decrease in the Siah-1 mRNA and protein levels in HPV-positive cells that are E6-mediated, avoiding β-catenin degradation. However, the activation of Wnt/β-catenin by the E7 oncoprotein is currently poorly understood [5].

An *in vitro* study showed that the HR and LR-HPV E6 proteins can distinctly augment the TCF response, with the highest activity observed for the HR-HPV E6 proteins [137]. In contrast to the previous research, E6 augmented the Wnt/β-catenin/TCF signaling response, although it did not significantly alter β-catenin stability and expression. This process did not depend on p53 degradation, the E6 PDZ-binding motif, APC/Axin/GSK3β complex activity, or β-catenin nuclear localization; instead, the presence of the E6/E6AP complex enhanced the TCF transcriptional activity mediated by the proteasome, independent of changes in β-catenin levels.

Subsequently, E6AP was confirmed to act as a novel Wnt signaling regulator that cooperates with E6 [138]. Specifically, the levels of E6 decrease in a proteasome-dependent manner in cells in which the Wnt pathway is activated; however, E6 is restored and stabilized in the presence of E6AP, suggesting that E6 requires E6AP to function in Wnt-activated cells. The participation of E6AP in the induction of the TCF response is independent of its catalytic activity. In contrast, β-catenin is stabilized by E6/E6AP, a process that requires the E6AP catalytic domain. Subsequently, β-catenin nuclear accumulation depends on its phosphorylation by GSK3β [138]. The mechanism by which E6/E6AP stabilizes β-catenin is not clear. To date, a direct interaction of E6 or E6AP with β-catenin has not been proven, but the E6/E6AP complex could alternatively participate in the sequestration of a negative regulator of the Wnt pathway.

Another element participating in the Wnt signaling pathway is FOXM1, which is also regulated by the HPV E6 oncogene. FOXM1 can induce β-catenin nuclear translocation by directly binding to β-catenin [58]. In cells harboring the HPV genome, E6 but not E7 oncoprotein was associated with FOXM1 overexpression [128]. This regulation is mediated by the MZF1/NKX2-1 transcriptional factors axis. E6 induces MZF1 expression, and MZF1 consequently activates NKX2-1 transcription. Because the FOXM1 promoter contains three putative sites for NKX2-1, E6 indirectly enhances FOXM1 transcription. In E6-expressing cells, the high levels of FOXM1 increase β-catenin translocation, which promotes TCF transcriptional activation and the expression of Wnt/β-catenin targets such as c-Myc and Cyclin D1 and stemness genes such as Nanog and Oct4. Thus, via the MZF1/NKX2-1 axis, E6 is responsible for metastasis, invasiveness, and stemness induced by the FOXM1-mediated activation of the Wnt/β-catenin pathway.

*In vivo* studies of transgenic mice support the role of the HPV E6 oncogene in the Wnt signaling pathway. In the K14E6 transgenic mice, the nuclear accumulation of β-catenin depends on the E6 PDZ-binding motif [102]. In this model, Wnt target genes (MYC, BIRC5, and CCND1) were up-regulated in the presence of full-length E6, but these genes were not up-regulated in mice expressing a truncated version of E6 lacking the PDZ-binding motif. Nevertheless, *in vitro* studies showed that both E6 forms enhanced TCF transcriptional activity due to the interaction of E6 with Dvl2, which is responsible for the disassembly of the β-catenin degradation complex [102]. These results suggest that the ability of E6 to activate the TCF response can be both dependent and independent of β-catenin translocation.

Other assays of double-transgenic mice expressing E7 and a constitutively active β-catenin indicated that the co-expression of both proteins promotes invasive cervical cancer, supporting that the activation of the Wnt/β-catenin pathway in premalignant lesions may be partly due to HPV [135].

**Figure 2:**
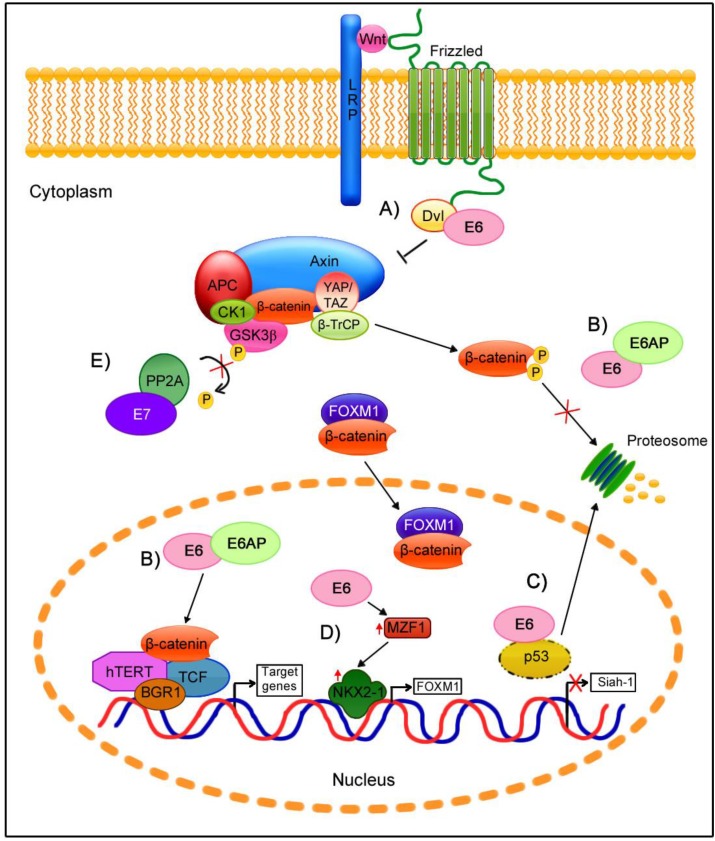
Participation of HPV oncogenes at different levels of Wnt/β-catenin cell signaling regulation. (**A**) The binding of E6-Dvl can disrupt the β-catenin degradation complex, releasing β-catenin which then accumulates in the cytoplasm; (**B**) The E6/E6AP complex stabilizes β-catenin, avoiding its proteasomal degradation and promoting its nuclear translocation, which results in an increase in TCF transcriptional activity; (**C**) E6-induced p53 degradation inhibits Siah-1 expression, which reduces β-catenin degradation; (**D**) E6 induces FOXM1 expression via the MZF1/NKX2-1 axis, which promotes FOXM1/β-catenin nuclear translocation and TCF transcriptional activation; (**E**) E7 binds to PP2A in the structural and catalytic domain, which may avoid the GSK3β activation and consequently β-catenin is stabilized.

Although the role of the E7 oncoprotein in the regulation of Wnt signaling has not been studied as well as that of E6, some findings suggest that this protein is involved in this pathway. Specifically, PP2A participates as a negative regulator of Wnt signaling. This phosphatase induces GSK3β activation via its dephosphorylation at the Ser 9 residue, which results in β-catenin degradation [139]. In a model based on primary human foreskin keratinocytes immortalized with E6 and E7 oncoproteins and transformed with SV40 small T antigen (smt), the smt antigen directly binds to the PP2A catalytic domain, preventing its activation to consequently induce Wnt signaling [75]. Moreover, cell line studies have demonstrated that the functions of E7 and smt are similar: they both strongly bind to the catalytic subunit of PP2A to inhibits its activity [114]. This role of E7 may contribute to β-catenin stabilization in the cytoplasm.

Notably, the above-described findings strongly support a role for HPV oncoproteins in the Wnt canonical pathway. Nevertheless, HPV has not been conclusively linked to the Wnt non-canonical pathway regulation, although some E6 targets, such as WNT7A and Dvl, are known to participate in the activation of Wnt non-canonical pathways.

Evidence supporting the role of HPV in the modulation of the Wnt signaling pathway is shown in Figure 2, which depicts the possible contribution of HPV oncoproteins at different levels in the activation of Wnt signaling.

## 7. Conclusions

Persistent infection with high-risk HPV types is clearly a main factor in cervical cancer development, and such infections are also implicated in the development of other types of cancer. HPV infection and the activation of diverse cellular processes such as signaling pathways are required to induce a malignant phenotype. Moreover, the Wnt/β-catenin pathway is deregulated in various neoplasias, and has been implicated in HPV-related cancers.

Several studies support the role of HPV oncoproteins in the activation of the canonical Wnt/β-catenin pathway, which may be involved in the onset, progression and maintenance of transformed cells.

Deciphering the precise mechanisms by which HPV oncogenes participate in Wnt/β-catenin modulation will help to elucidate HPV-related carcinogenesis. This information could eventually aid in identifying biomarkers of prognosis and contribute to the design of more effective targeted therapeutics.
